# Advanced microstructure imaging at high b‐values and high resolution combining ultra‐high performance gradient diffusion imaging and model‐based deep learning demonstrated using 3D multi‐slab acquisition

**DOI:** 10.1002/mrm.70046

**Published:** 2025-08-24

**Authors:** Chu‐Yu Lee, Reza Ghorbani, Mahsa Rajabi, Merry Mani

**Affiliations:** ^1^ Department of Radiology University of Iowa Iowa City Iowa USA; ^2^ Department of Electrical and Computer Engineering University of Virginia Charlottesville USA; ^3^ Department of Radiology and Biomedical Engineering University of Virginia Charlottesville Virginia USA

**Keywords:** 3D k‐space acceleration, 3D multi‐slab diffusion‐weighted imaging, compartment models, high‐performance gradients, MAGNUS, model‐based deep learning, multi‐dimensional diffusion encoding, plug‐and‐play priors, q‐space trajectory imaging (QTI)

## Abstract

**Purpose:**

To demonstrate the extended capabilities of 3D multi‐slab diffusion‐weighted acquisition (3D‐msDWI) on high‐performance gradients (HPG) to support advanced microstructure modeling for in‐vivo human studies at high resolutions.

**Methods:**

Despite optimal SNR‐efficiency, the application of 3D‐msDWI has been limited by the long volume acquisition times (VAT) required for encoding the 3D k‐space using multi‐shot approaches. Substantial reduction of VAT is possible by employing optimized 3D k‐space under‐sampling methods. We demonstrate that with reduced VAT, 3D‐msDWI can be successfully utilized for advanced brain microstructure modeling at high resolution. HPG systems (e.g., >200 mT/m, >300 T/m/s) enable further optimization through shorter echo times at high b‐values. We evaluated the accelerated 3D‐msDWI method's ability to support diffusion studies at 1mm isotropic resolution using data collected across three shells, with b‐values extended up to 6000 ${\text{s/mm}}^{2}$, and employing compartment models. The reconstruction employed a navigator‐based, motion‐compensated approach using a regularized, iterative model‐based algorithm.

**Results:**

The accelerated 3D‐msDWI framework enabled the generation of whole‐brain parametric maps of a three‐compartment model, at 1mm isotropic resolution, using a 3‐shell, 66‐direction acquisition completed in <15 min. The intra‐axonal diffusivities (in $\mu m^{2}/ms$) and volume fractions reported from the method are as follows: 2.27 ± 0.14; 0.6 ± 0.04 in corpus‐callosum, 2.17 ± 0.09; 0.66 ± 0.03 in anterior limb of internal capsule, 2.18 ± 0.08; 0.68 ± 0.04 in posterior limb of internal capsule, 2.07 ± 0.06; 0.62 ± 0.04 in corona radiata, 2.25 ± 0.08; 0.68 ± 0.04 in cortico‐spinal tract, 2.12 ± 0.04; 0.63 ± 0.05 in superior longitudinal fasciculus, with a coefficient of variation <10% across subjects for all regions studied. The quantified values were validated using standard single‐diffusion and multi‐dimensional q‐trajectory encoding acquisitions.

**Conclusion:**

The inherent optimal SNR‐efficiency of the 3D‐msDWI framework can be harnessed for whole‐brain high‐resolution advanced microstructure modeling for in‐vivo human studies, using advanced hardware and reconstruction.

## 1 INTRODUCTION

Diffusion‐weighted magnetic resonance imaging (dMRI) is a widely utilized non‐invasive tool to study the brain tissue microstructure by making use of the random diffusing motion of water molecules inherently present in living tissue. The exceptional sensitivity of dMRI to fine‐scale water displacement—extending well beyond the imaging resolution—coupled with advancements in mesoscale resolution, brings us closer to achieving the goal of in‐vivo histology.^1^, ^2^ In this approach, histological features at the microscopic level are inferred from mesoscopic MRI measurements through the use of tissue modeling and tailored acquisition protocols. In recent years, MRI hardware advances—such as improved slew rates—have enabled the acquisition of dMRI data at 1 mm isotropic voxel size using standard 2D single‐shot DW‐EPI sequences.^3^ Additionally, the improved maximum gradient strength (>200 mT/m) has enabled the acquisition of high b‐values at shorter echo times (TE).^3^, ^4^ Higher b‐values enable better sensitization of dMRI measurements to tissue histological features at the micron scale, thus improving the accuracy and precision of the tissue model fits from the measured data. At higher b‐values $>4000\;s/mm^{2}$, the diffusion signal is primarily sensitive to the intra‐axonal compartment, which enables the separation of diffusivity estimates of the intra‐ and extra‐axonal compartment of the tissue.^5^, ^6^, ^7^ This can have important implications, such as offering the potential to differentiate between specific intra‐ and extracellular disease processes.^6^

Single‐shot 2‐dimensional (2D) DW‐EPI remains the main workhorse for most high b‐value diffusion MRI applications on the advanced scanning platforms. One downside of this combination is the inherently low SNR of the diffusion contrast, which leads to imaging near the noise floor. Specifically, when higher b‐values are desired, this intrinsically low SNR regimen necessitates compromising the spatial resolution of the imaging even on HPG systems. A standard workaround is to employ multiple averages at the expense of long scan times. While multi‐shot 2D methods can improve in‐plane resolution of high b‐value scans, thick slices are typically resorted to maintain sufficient SNR. Additionally, the SNR‐efficiency of single‐shot and multi‐shot 2D methods is hampered by the long repetition times (TR) required in situations involving increased slice coverage.

3D multi‐slab diffusion MRI (3D‐msDWI) is known for its ability to achieve optimal SNR‐efficiency,^8^, ^9^ defined as the SNR per unit time. Here, the imaging is typically performed in a multi‐slab configuration, which is the key factor enabling the short TR for achieving whole‐brain coverage. 3D‐msDWI enables whole‐brain coverage, even at high spatial resolutions, using 10–20 slabs with a TR of ∼2 s, which is close to the optimal TR range of 1–1.6 s for 3T. In comparison, the 2D methods suffer from very long TRs (in the range of 8–15 s without multi‐band, or 3–6 s with multi‐band) as the number of slices increases.^10^ Due to its high SNR‐efficiency, 3D‐msDWI is an ideal candidate for achieving high isotropic resolution for high b‐value studies. However, 3D‐msDWI is associated with long volume acquisition times (VAT), due to its multi‐shot k‐space sampling along the $k_{z}$ dimension. VAT, the time required to fully sample a slab, is the product of the number of $k_{z}$ shots required to sample all $k_{z}$ frequencies (including over‐sampling, see^11^), and the TR. In the classical fully‐sampled setting, the VAT of the 3D‐msDWI, which is much longer than that for a 2D acquisition with matched slices.^10^ The high VAT inherent to standard implementations of 3D‐msDWI poses a challenge for its use in in‐vivo human studies, limiting its perceived impact and applicability.

Recently, several advanced k‐space sampling strategies were developed to improve the volume acquisition efficiency of 3D‐msDWI methods. Li et al.^12^ proposed a k‐space trajectory to sample 2 $k_{z}$ encodes per TR, and later proposed a self‐navigated sampling where multiple kz frequencies are sampled during each shot.^13^ Lee et al.^11^, ^14^ proposed a more aggressive k‐q acceleration employing a 2D CAIPI $k_{y}$‐$k_{z}$ under‐sampling randomized across volumes to achieve >3× reduced VAT. All of the above advanced sampling schemes are driven by regularized, iterative model‐based reconstruction techniques, which utilize either hand‐crafted priors^12^, ^13^ or deep‐learned (DL) priors^11^, ^14^ during reconstruction to handle the sampling‐specific reconstruction artifacts. Recently, T‐hex sampling^15^ was proposed to accelerate 3D‐volumetric acquisition by replacing the multi‐shot sampling with single‐shot sampling. Importantly, the ability to optimize the sampling pattern along both phase‐encoding dimensions of the 3D k‐space is opening up a window to shorten the VAT to make 3D‐msDWI a practical and superior alternative to 2D methods for in‐vivo human applications.

Accelerated 3D‐msDWI with reduced VAT helps to trade the scan time for increased q‐space coverage. Extending accelerated 3D‐msDWI to HPG systems provides an additional SNR boost from shorter TE. This allows the exploration of 3D‐msDWI for a high b‐value regimen for the first time for in‐vivo human studies. In this work, we evaluate the potential of combining 3D‐msDWI with high b‐value acquisition to enable advanced microstructure modeling at high resolutions. We evaluate the VAT‐accelerated 3D‐msDWI implementation in Reference [11] for whole‐brain 1‐mm isotropic resolution imaging in the context of 3‐compartment modeling^16^, ^17^, ^18^, ^19^ which requires high b‐value shells to separate signals from the intra‐ and extra‐axonal compartments. The 10× under‐sampled slab data is reconstructed using a 3D‐qModeL reconstruction.^11^, ^20^, ^21^ We test the validity of the above acquisition‐reconstruction framework by comparing the model parameters with 2D single‐shot methods acquired at a lower resolution and using region‐based analyses. Specifically, we compare the microstructural parameters obtained from white‐matter (WM) regions‐of‐interest (ROIs) of the accelerated 3D‐msDWI framework to those obtained from the 2D methods, such as: (i) a standard 2D multi‐shell acquisition, and (ii) a 2D multi‐dimensional diffusion encoding acquisition.^22^ The preliminary work in this report provides evidence to support further exploration of accelerated 3D‐msDWI as a viable scheme for high‐resolution microstructure studies requiring extended k‐q space coverage for in‐vivo human applications.

## 2 METHODS

Our goal is to demonstrate the feasibility of supporting the 3‐compartment modeling at high resolution using the VAT‐accelerated 3D‐msDWI. This model separately accounts for the intra‐ and extra‐axonal contributions to the diffusion signal S(b,g) as:

(1) $$S(b,g)=S_{0}\int_{\text{n}}d\text{n}𝒫(\text{n})⊛K(b,\text{g}\cdot n)$$

where $K(b,g\cdot n)=\underset{\text{intra‐axonal}\;}{\underbrace{f_{a}e^{-bD_{a}\text{g}\cdot n}}}+\underset{\text{extra‐axonal}}{\underbrace{f_{e}\;e^{-bD_{e}^{⊥}-b(D_{e}^{\|}-D_{e}^{⊥})\text{g}\cdot n}}}+\underset{\text{CSF}}{\underbrace{f_{w}e^{-bD_{w}}}}.$

We aim to estimate the model parameters: $f_{a}$, $D_{a\;}$, $D_{e}^{\|}$, $D_{e}^{⊥}$ and $f_{w}$ from an accelerated 3D multi‐shell acquisition (more details related to the model and estimation methods are given in Supporting Information Subsection A). Towards this, we design our diffusion protocol to have 3 shells at b‐values of 1000, 2000, and 6000 ${\text{s/mm}}^{2}$, with 22 samples on each shell, resulting in a total of 66 directions. The shells are chosen such that the lower shells support a diffusion tensor (DTI) and diffusion kurtosis (DKI) fitting, while the higher b‐values will contribute to the accuracy of the intra‐neurite parameter estimates, and the lower b‐values will contribute to the accuracy of the extra‐neurite parameter estimates.^19^

**FIGURE 1:**
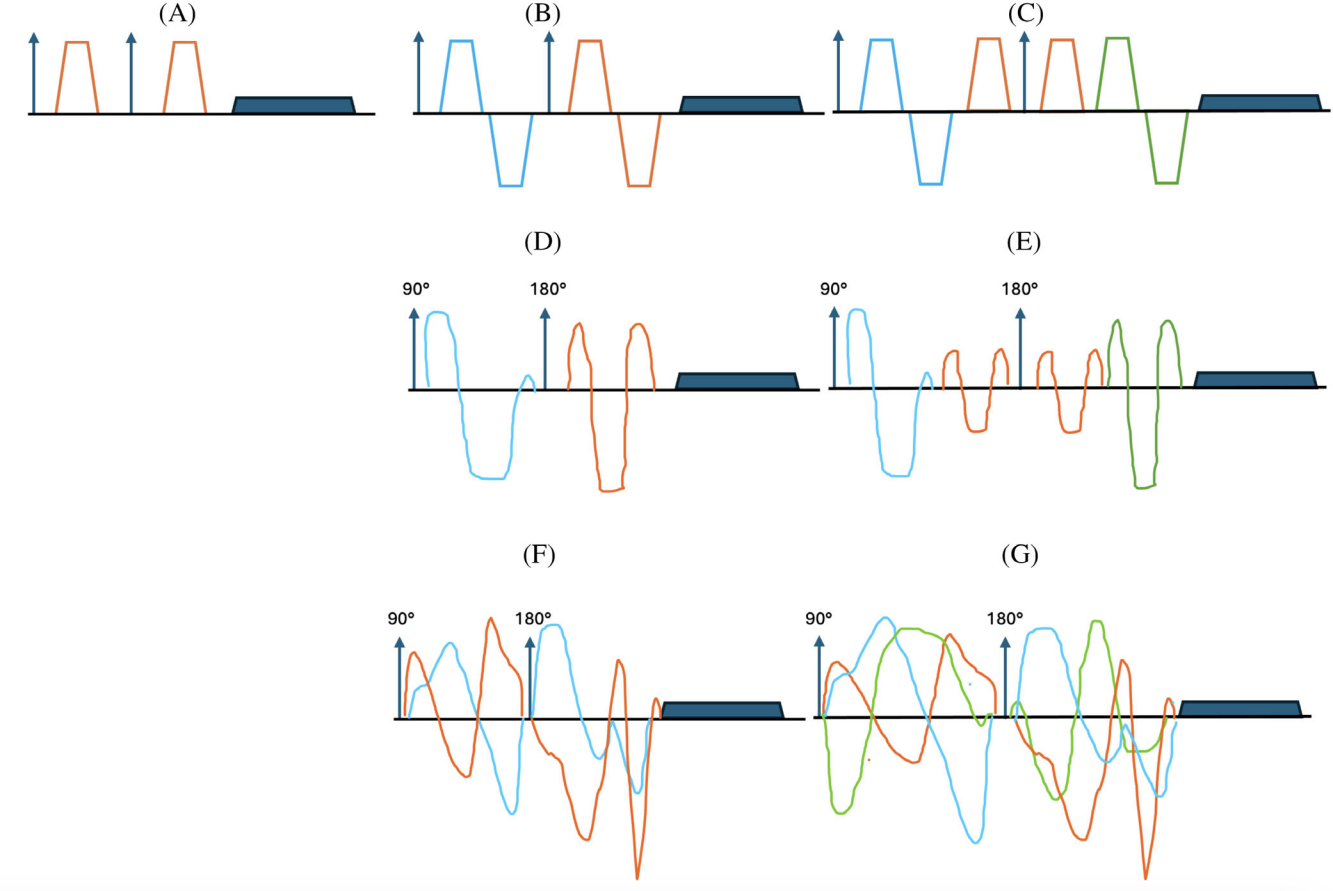
Illustrative example of SDE and MDE: (A) In SDE, diffusion encoding along one direction per signal preparation and readout is employed. While SDE encodes a rank‐1 tensor, it typically refers to the pulsed field gradient implementation as shown above. In MDE, multiple diffusion encoding directions per signal preparation and readout are employed (denoted by gradients in different colors). Here in (B,C), two specific cases pertaining to planar, spherical encodings are shown as published in References [23, 24, 25]. MDE can be achieved using pulsed field gradients as in (B,C), or using arbitrary gradients as shown in (D–G). Arbitrary gradient shapes allow the design of specific B‐tensors as desired. Recent implementations make use of overlapping arbitrary gradients, which are more efficient as shown in (F,G).^25^

Previous works have utilized standard 2D‐based single‐diffusion encoding (2D‐SDE) schemes, and more advanced multi‐dimensional diffusion encoding (2D‐MDE) schemes^22^, ^26^, ^27^ for the parameter estimation of the above model. We compare the microstructure parameter maps derived from the 3D‐qModeL reconstruction to those obtained from the 2D‐SDE and 2D‐MDE methods. SDE is the conventional method for acquiring diffusion data using pulsed field gradients (PFGs). Here, the diffusion signal is sensitized to a linear gradient applied along a single direction using trapezoidal gradient shapes. In contrast, MDE makes use of multiple diffusion encoding directions simultaneously. MDE thus provides improved sensitivity to higher‐order moments of the diffusion process^22^, ^26^, ^27^ and can provide better separability of the compartmental diffusivities and volume fractions. MDE approaches such as q‐trajectory imaging (QTI) replace the PFG with time‐varying gradients.^22^, ^26^, ^27^ In addition to providing better encoding efficiency,^28^ it allows the design of waveforms that can probe the response to a desired ellipsoid encoded by a 3×3 B‐tensor. Figure 1 provides an illustration of the SDE and the MDE waveforms. A recent study by^19^ explored the optimal QTI protocol for the 3‐compartment model fitting, using a modified kernel to account for the B‐tensor encoding:

(2) $$\begin{array}{ll} K(b,\beta ,\hat{g}\cdot n) & =f_{a}e^{-bD_{a}(\beta (\hat{\text{g}}\cdot n-\frac{1}{3})+\frac{1}{3})}\\ & \;+f_{e}\;e^{-bD_{e}^{⊥}-b(D_{e}^{\|}-D_{e}^{⊥})(\beta (\hat{\text{g}}\cdot n-\frac{1}{3})+\frac{1}{3})}+f_{w}e^{-bD_{w}} \end{array}$$

where β accommodates the diffusion encoding shape of the QTI measurements. In this study, we make use of the optimized QTI‐protocol to derive the 3‐compartment model parametric maps using the 2D‐MDE methods. With more extensive q‐space coverage and a richer diffusion encoding, the MDE parameter maps will serve as an independent validation for the 3D‐msDWI‐derived parameter maps.

### 2.1 Experiments and datasets

Data were collected from 9 healthy subjects (4 adults in the age range of 30–50 years and 5 older adults in the age range of 70–80 years), using a 32‐channel NOVA head‐coil. All studies were approved by the Institutional Review Board at the University of Iowa.

#### 2.1.1 3D‐SDE

We implemented the accelerated 3D‐msDWI acquisition on the 3T MAGNUS scanner at the University of Iowa (G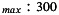 mT/m; SR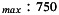 T/m/s) using KS Foundation.^29^ The standard PFG‐based SDE implementation was utilized. The actual maximal gradient strength and slew rate used for the diffusion encoding and readout gradients are 270 mT/m and 244 T/m/s, respectively. Eight axial slabs were prescribed to cover the whole brain (112 mm coverage along the slice direction). Each slab thickness was 14 mm and was extended to a 20‐mm FOVz (40% over‐sampling). To reduce VAT, we utilized the 2D CAIPI $k_{y}$‐$k_{z}$ under‐sampling by a factor of ∼10 (Ry = 3, Rz = 3.33),^11^ and randomized the under‐sampling pattern for each DW volume. On the HPG, a TE of 52 ms is achieved for the maximum b‐value of 6000 ${\text{s/mm}}^{2}$. With a TR of 2 s, 6 $k_{z}$ encoding over a 20‐mm FOVz was designed to provide 20 1‐mm thick slices in 12 s per diffusion volume. Within each TR, a 2D navigator was also acquired. The shortened VAT allows collection of 66 DW volumes at 1 mm isotropic resolution in a scan time of 13.8 min, including a fully‐sampled non‐DW volume.

#### 2.1.2 2D SDE

For validation purposes, whole‐brain SDE data with matched scan time and q‐space coverage were also collected on all subjects. A sagittal single‐shot 2D‐DW‐EPI with a multi‐band acceleration of 3 and a NEX of 3 was utilized. In a scan time of 15.18 min, 66 DW and 3 non‐DW volumes were collected over three shells, albeit with a lower spatial resolution of 1.5 mm isotropic. More details about the imaging parameters are provided in Table S1 in the Supporting Information.

#### 2.1.3 2D MDE

Additionally, whole‐brain MDE data were collected using the optimized QTI protocol.^19^ The MDE waveforms are designed using the NOW toolbox^25^, ^28^ and were optimized for the best performance of the MAGNUS gradients, where the NOW‐optimized waveforms report a gradient strength utilization of 270 mT/m. The 2D‐MDE protocol (see Table 1) utilized of three B‐tensor shapes corresponding to β = 1, 0.7 and 0. The total scan time was 15.03 min to acquire 1.5 mm isotropic resolution data using a sagittal single‐shot 2D acquisition with a multi‐band acceleration of 3. Please refer to Table S1 in the Supporting Information for more details about the imaging parameters.

**TABLE 1:** Details of models and encodings used.

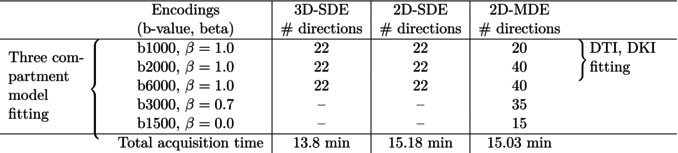

### 2.2 Reconstruction and processing

#### 2.2.1 3D‐msDWI

The 10× under‐sampled 3D data of each slab were reconstructed using the 3D‐qModeL reconstruction using an off‐line pipeline as follows: The 3D coil sensitivities and slab‐profiles for each slab were calibrated from the fully sampled non‐DW scan. The under‐sampled 2D navigator was reconstructed using an iterative CG‐SENSE reconstruction for each $k_{z}$ shot of the imaging.^11^ The phase estimated from the navigator was used in the 3D‐qModeL reconstruction,^11^ which jointly reconstructs all 66 DW volumes while also achieving phase‐compensation. The 3D‐qModeL reconstruction is formulated as:

(3)

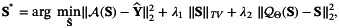


Here S is the multi‐directional 4D‐DW volume to be reconstructed, and $\hat{Y}$ is the measured multi‐channel k‐space data from all directions, under‐sampled using the randomized 2D CAIPI scheme. The forward model, 𝒜, denotes a 2D‐SENSE reconstruction with phase‐compensation for the reconstruction of the 2D $k_{y}$‐$k_{z}$ CAIPI accelerated 3D volume.^11^ The second term in Equation [3] is a spatial total‐variation regularizer,^30^ and the third term is a q‐space regularizer that minimizes the $\ell_{2}$ error along the q‐dimension to enable the joint k‐q reconstruction. Here, ${𝒬}_{\Theta }$ represents a plug‐and‐play denoising auto‐encoder (DAE) network with parameters Θ, that is trained to denoise the q‐space signal of a predetermined q‐space. This DAE network consisted of a compact 3‐layer, fully connected auto‐encoder, and is trained to denoise the signal of the anticipated q‐space in the current experiment, i.e., 3 shells with b‐values of $1000,2000,6000\;{\text{s/mm}}^{2}$. The training was performed using simulated data that were generated to match the anticipated q‐space given above.^20^, ^21^ The DAE training employed a weighted mean‐squared error (MSE) loss function, where a higher weight on the MSE of the b6000 shell was enforced. We observed that the higher weight helps to lower the MSE of the b6000 shell as well as other shells, which in turn improves the robustness of the parameter estimation. Table S2 in the supporting Information lists the losses from each shell using the different weights tested, as well as the total loss. Based on this, we chose a weight of 1.5 for the b6000 shell. For comparison, the under‐sampled 3D data was also reconstructed using the ablated qModel reconstruction (λ_2_ is set to zero).

After data from all slabs were reconstructed using 3D‐qModeL separately, the slabs were combined using^31^ to generate whole‐brain diffusion volumes. The reconstructed volumes were utilized for 3‐compartment model fitting using the Standard Model Imaging (SMI) toolbox^19^ after brain extraction using BET.^32^ No further preprocessing of the qModeL reconstructed data was done prior to model fitting.

#### 2.2.2 2D‐SDE and 2D‐MDE

The 2D‐SDE and 2D‐MDE data were pre‐processed using Marchenko‐Pastur Principal Component Analysis (MPPCA) denoising,^33^ followed by motion‐correction and eddy current correction.^34^ The 3‐compartment model fitting was performed using the SMI toolbox using data from all shells. DTI and DKI fitting were also performed on these datasets using data from the b1000 and b2000 shells with linear encodings. Table 1 lists the encoding details and the shells used for fitting the various models.

ROI‐based approaches were utilized to study the validity of the parameter maps derived from the 3D‐msDWI framework. Six WM ROIs were selected from the JHU white‐matter atlas^35^: Corpus callosum (CC), anterior limb of the internal capsule (ALIC), posterior limb of the internal capsule (PLIC), corona radiata (CR), superior longitudinal fasciculus (SLF), and cerebral peduncle (CP). The reconstructed parameter maps from all subjects were registered to the atlas space using FSL's FNIRT non‐linear registration,^36^ and parameter values corresponding to all three reconstructions were extracted from the ROIs.

## 3 RESULTS

**FIGURE 2:**
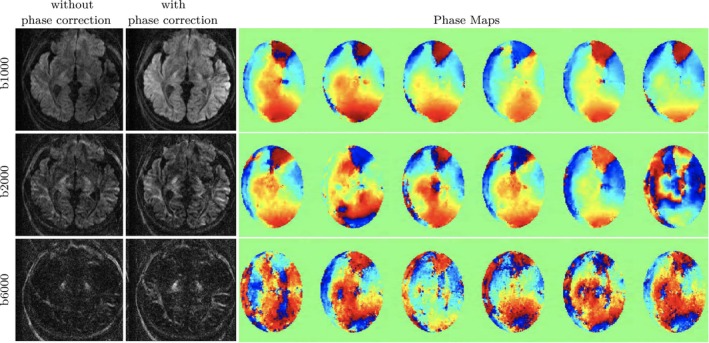
Reconstructions with and without phase correction are shown from the accelerated 3D‐msDWI data. The phase maps estimated from the 2D navigator for the 6 kz planes sampled per slab are shown for the three b‐values of a given diffusion direction.

**FIGURE 3:**
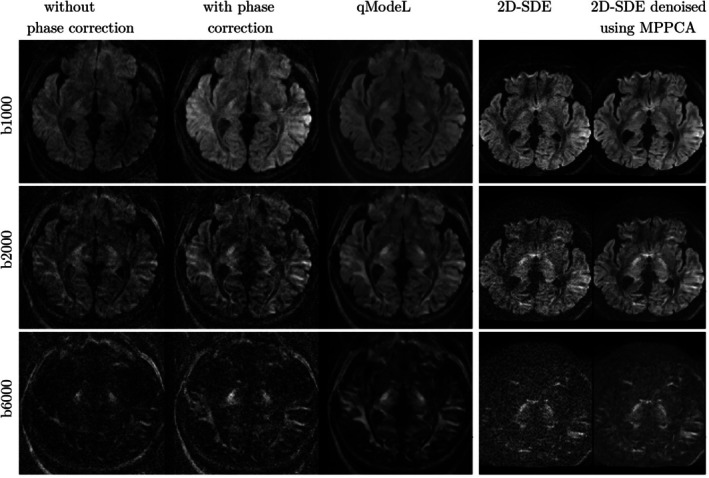
Elaborate comparison of phase compensation and denoising example from subject #1. To evaluate the efficacy of the navigator‐based phase compensation in the 3D‐msDWI reconstruction, the corresponding 2D‐SDE images and their denoised versions are provided. (Note that the displayed FOVs of the 3D and 2D datasets are different due to 3D being an axial scan and 2D being a sagittal scan. As a result, the distortions in the 2D‐SDE denoised images are not perfectly matched. However, they still serve to confirm that phase compensation in the 3D‐msDWI qModeL reconstruction.) The qModeL images are noted to be devoid of phase artifacts (third column). Further, the qModeL images are also denoised. For comparison, the MPPCA denoised 2D SDE images are also shown in the last column.

We first investigate the ability of the 2D navigator‐based reconstruction to compensate for the phase errors at the high b‐value for the accelerated 3D‐msDWI. At the b‐value of 6000 ${\text{s/mm}}^{2}$, a lower SNR is expected for the imaging and the navigator echo compared to the lower b‐shells. Figure 2 shows the phase maps generated from the under‐sampled navigator, as well as the phase‐compensated reconstruction from the three b‐shells of a given slice. The estimated phase maps for the six $k_{z}$ planes sampled per slab, for all diffusion directions and b‐shells, are utilized in the 3D‐qModeL phase‐compensated joint reconstruction, which reconstructs all the 66 volumes simultaneously per slab. The qModeL images from a representative subject are provided in Figure 3. These images show adequate phase compensation as well as denoising. This can be evaluated by comparing with the corresponding 2D‐SDE images and their denoised versions provided in Figure 3. Figure S1 in the Supporting Information shows the phase compensation and denoising from a second subject. Figure S2 in the Supporting Information shows the whole‐brain reconstruction of the qModeL images after slab‐combination.

**FIGURE 4:**
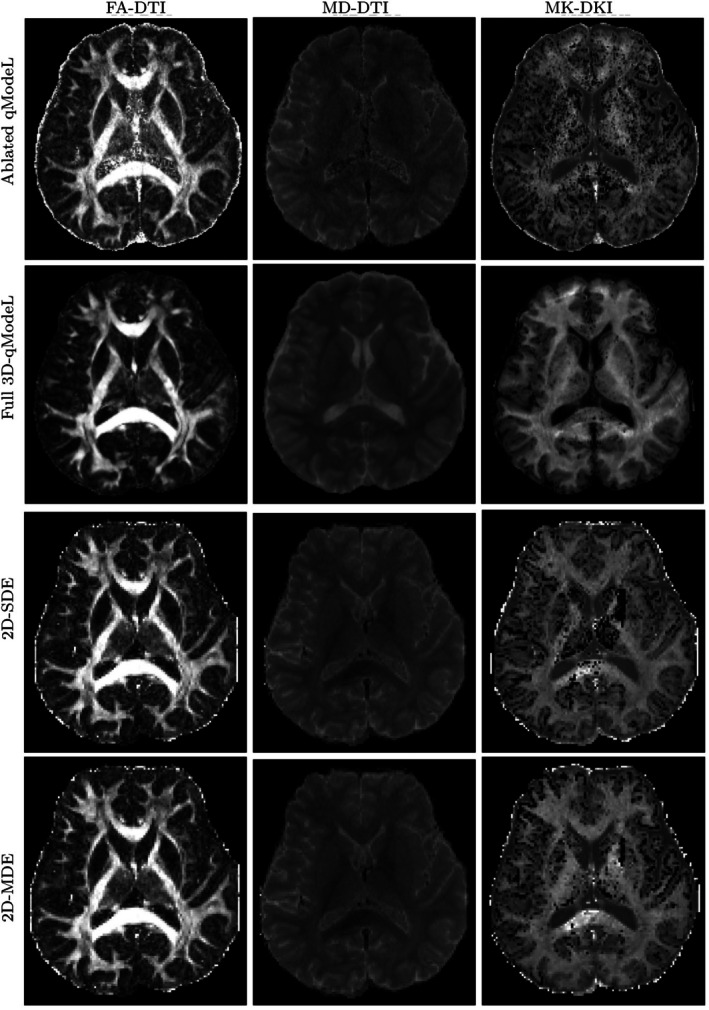
FA, MD, and MK maps computed for the various acquisitions. The ablated qModeL and the full qModeL maps are of 1 mm isotropic resolution, whereas the 2D‐SDE and 2D‐MDE are of 1.5 mm isotropic resolution. These maps were estimated from diffusion data acquired on the lower shells (corresponding to b1000 and b2000). A band of abnormal MD and MK values is noted in the 3D‐qModeL MD and MK maps, which corresponds to regions with inadequate fat suppression in the 3D acquisition. Please see Figure S3 in the supporting information for the same maps from a second subject.

Figures 4 and S3 show the DTI and the DKI model fits from two subjects: Fractional anisotropy (FA‐DTI), mean diffusivity (MD‐DTI), and mean kurtosis(MK‐DKI) estimated from the b1000 and b2000 shells of the three acquisitions. The first row corresponds to parameter maps estimated from an ablated qModeL reconstruction where the q‐space regularization using the DAE was not utilized. The second row corresponds to the full qModeL reconstruction with q‐space regularization. The third and fourth rows show the parameter maps estimated using the 2D‐SDE and 2D‐MDE data, respectively. A comparison of these maps shows that the parameters match across the acquisitions, although the ablated qModeL maps are noisy, as expected. The MK map is less noisy from the 3D‐qModeL reconstruction, likely from the joint regularized recovery that enforces spatial and q‐space continuity, resulting in fewer voxels with spurious values outside the biophysical range. DKI fits are typically hampered by unstable fits that result in “black holes.” This is noted in the 2D‐SDE and 2D‐MDE fits, while mostly absent in the 3D‐qModeL fits. Additionally, MD values in the CSF region were notably different across different acquisitions and reconstructions, likely due to different SNR levels. Nontheless, the general agreement of the 3D‐qModeL parameter maps with the 2D maps within gray/white matter confirms that the accelerated 3D‐msDWI data is reconstructed with sufficient quality using the 3D‐qModeL reconstruction, especially for the data from the lower shells.

**FIGURE 5:**
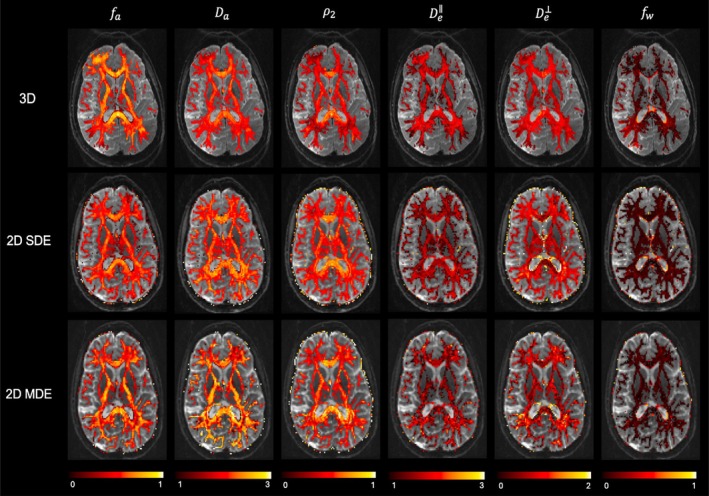
Three‐compartment model parameters shown from the white matter regions from all three acquisitions (from a subject belonging to the younger adult group). The diffusivities are displayed in the units $\mu m^{2}/ms$.

**FIGURE 6:**
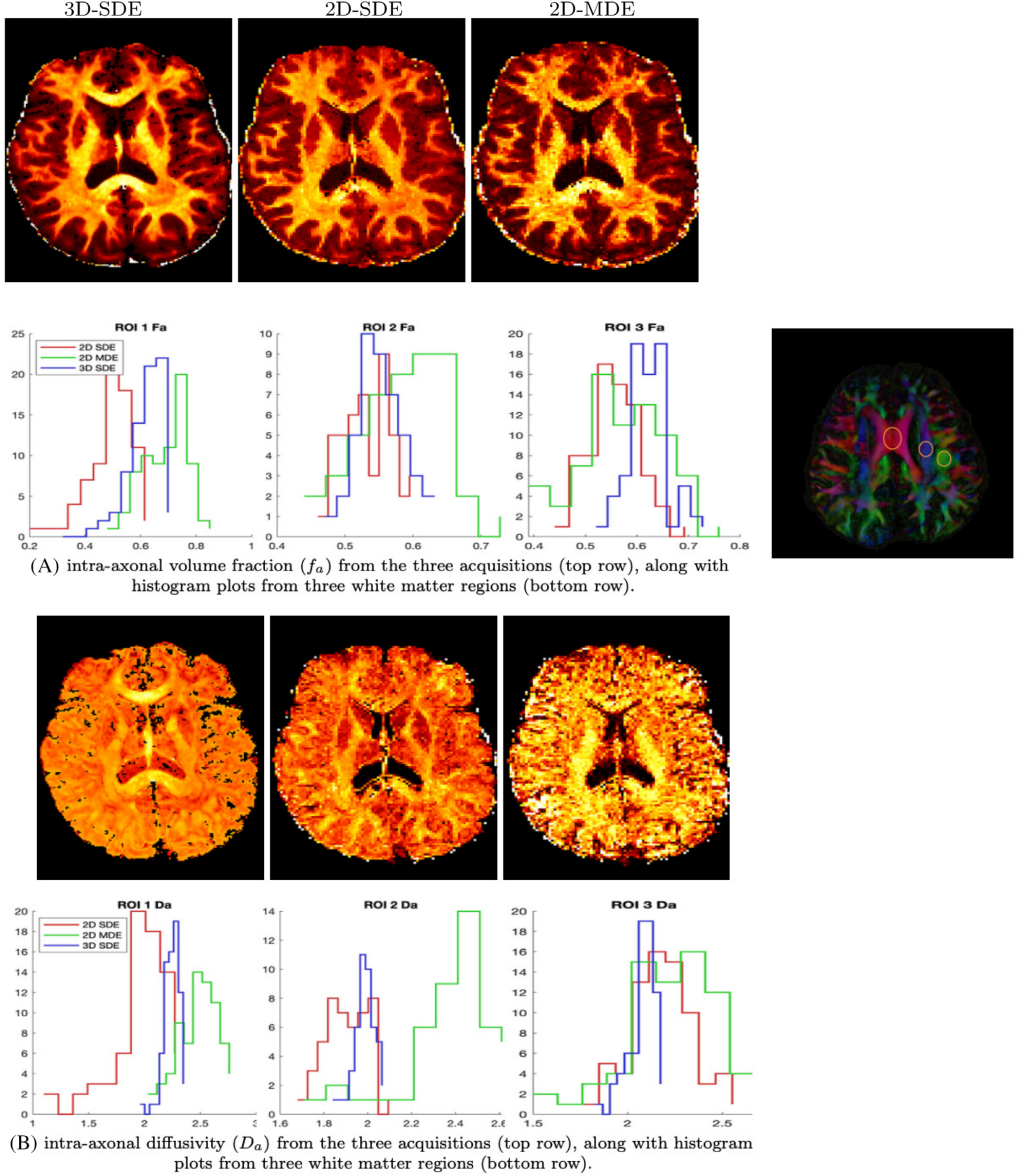
Comparison of intra‐neurite parameter values from three white matter ROIs across the three acquisitions. The ROIs are marked on the RGB map in orange circles.

**FIGURE 7:**
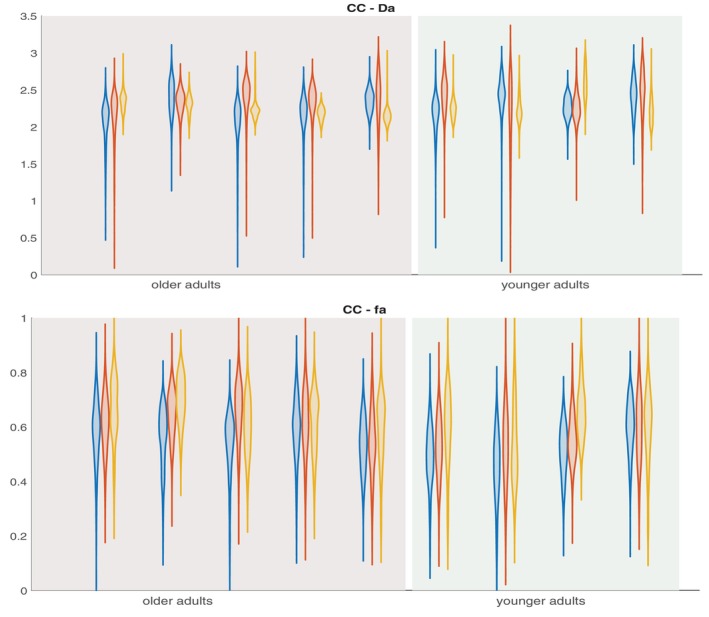
Plots of the distribution of $D_{a}$ and $f_{a}$ values from the CC white matter ROI from all 9 subjects. Here blue, red and yellow colors represent 2D‐SDE, 2D‐MDE, and 3D‐SDE, respectively.

Figures 5, 6, 7 show the parameter maps of the 3‐compartment model from all three acquisitions. Figure 5 shows a comparison of all the parameters from a younger subject, where the overall range of parameters is noted to agree across the acquisitions. The parameter maps from an older subject are provided in Supporting Information (Figure S4). Given the high sensitivity of 3‐compartment model fitting to noisy perturbations, the close agreement between the 3D‐qModeL fits and the 2D fits indicates adequate performance of the accelerated 3D‐qModeL framework. Figure 6 shows a closer analysis using histogram plots for the parameters $f_{a}$ and $D_{a}$ (in $\mu m^{2}/ms$) from three WM ROIs. The histogram plot confirms that the range of parameters estimated from the 3D‐qModeL overlaps with the range of parameter values obtained from the 2D‐SDE and 2D‐MDE acquisitions with traditional reconstructions at moderate accelerations. Moreover, the conformance of whole‐brain maps across the 2D and 3D images confirms that the 3D‐qModeL regularizer is trained in the appropriate range for recovering the parameter maps from all brain voxels and not just the WM voxels, which is crucial for recovery using joint k‐q approaches.

**FIGURE 8:**
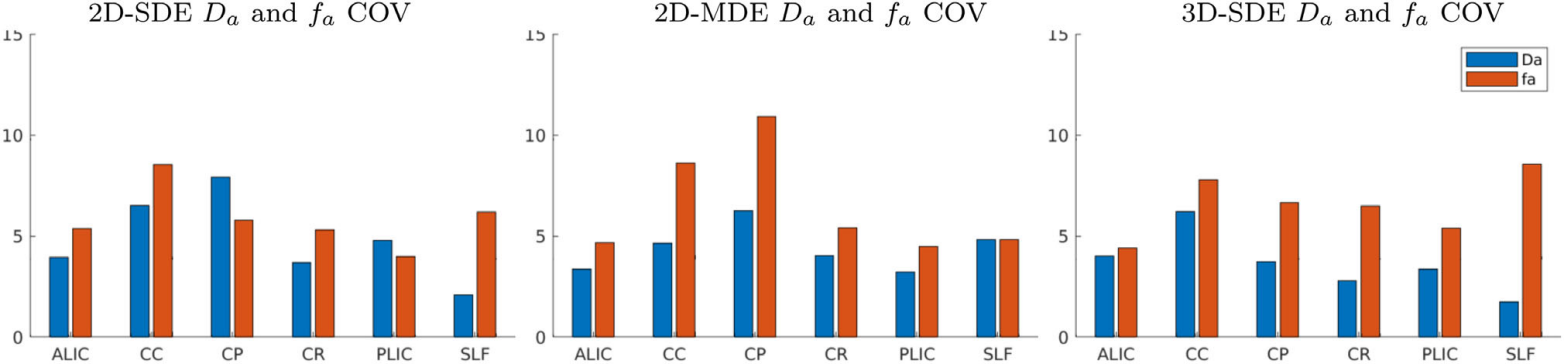
Mean, standard deviation, and COV from ROIs across subjects for the different methods.

In addition to the above subject‐level evaluations, we performed across‐subject evaluations of the parameter estimates using an atlas‐based ROI approach. We plotted the summary of the $f_{a}$ and $D_{a}$ parameter values estimated from the WM ROIs from all the subjects using a violin plot that shows the distribution of the $f_{a}$ and $D_{a}$ across all methods. In Figure 7, the $f_{a}$ and $D_{a}$ parameter values observed from the CC ROI are shown as a violin plot; the plots from the other ROIs are added in the Supporting Information Figures S5–S6. From these figures, the distribution of the parameter values is noted to be consistent across the three acquisition methods for all the subjects included in the study. Table 2 lists the mean and standard deviation of the $f_{a}$ and $D_{a}$ parameters from the six WM ROIs across all 9 subjects. The corresponding values estimated from the 2D‐SDE and 2D‐MDE datasets are also reported in the table, which shows good agreement with the 3D‐qModeL estimated values. We also plot the variability in the range of parameters across subjects by quantifying the coefficient of variation (COV) across subjects. The COV plot across subjects is included in Figure 8. The COV is noted to be <10% for all ROIs for the 3D‐SDE acquisition, and is comparable to the COV noted for the 2D methods.

**TABLE 2:** Mean and standard deviation across subjects of $f_{a}$ and $D_{a}$ from six WM ROIs for the different methods.

Mean and standard deviation of $D_{a}$ (in μm^2^/ms)				Mean and standard deviation of $f_{a}$		
	2D‐SDE	2D‐MDE	3D‐LTE	2D‐SDE	2D‐MDE	3D‐LTE
CC	2.2441 ± 0.1461	2.2784 ± 0.1063	2.2679 ± 0.1409	0.5399 ± 0.0461	0.5823 ± 0.0502	0.5972 ± 0.0466
ALIC	2.1336 ± 0.0845	2.3570 ± 0.0794	2.1691 ± 0.0872	0.5745 ± 0.0308	0.6439 ± 0.0302	0.6589 ± 0.0291
PLIC	2.1415 ± 0.1026	2.4453 ± 0.0790	2.1813 ± 0.0736	0.6050 ± 0.0241	0.6918 ± 0.0311	0.6826 ± 0.0369
CR	2.1372 ± 0.0789	2.4695 ± 0.0996	2.0759 ± 0.0581	0.5718 ± 0.0304	0.6545 ± 0.0354	0.6188 ± 0.0402
CP	2.0191 ± 0.1598	2.2237 ± 0.1392	2.2503 ± 0.0838	0.6034 ± 0.0350	0.6931 ± 0.0757	0.6846 ± 0.0456
SLF	2.3083 ± 0.0484	2.3410 ± 0.1133	2.1231 ± 0.0372	0.6015 ± 0.0373	0.6035 ± 0.0291	0.6318 ± 0.0541

We also performed additional experiments to evaluate the raw images and validate whether the perceived SNR improvement at ultra‐high b‐values in the 3D‐qModeL reconstruction reflects true diffusion contrast. Validations were conducted using real and simulated data, and a separate high‐SNR reference data, with results provided in Supporting Information Figures S7–S10. In Figure S7, we identified a voxel in the b6000 image where a perceived SNR improvement is noted in the 3D‐qModeL reconstruction compared to the 2D‐SENSE reconstruction. In Figure S8, we show that the SNR improvement offered by the 3D‐qModeL reconstruction is at the expected level by comparing to a stable voxel (where no SNR improvement was noted) within the same anatomy, supporting the notion that the DAE does not alter the diffusion contrast. Here, the “denoising only” refers to the output of the DAE only (${𝒬}_{\Theta }(S)$ in Equation (3)), whereas the full 3D‐qModel reconstruction enforces consistency to the measured data as given in Equation (3). We further simulated the q‐space signal using the tissue parameters estimated for that voxel from both the 2D‐MDE and 3D‐qModeL fits. Both simulations closely predict the 3D‐qModeL signal at all b‐values, including the ultra‐high b‐value (shown in Figure S9). We further simulated the diffusion signal for all the voxels in the brain WM using the qModeL parameter estimates. Figure S10 shows the diffusion contrast from 5 gradient directions at the ultra‐high b‐value, which is compared to the 3D‐qModeL reconstruction. Additionally, in a separate experiment, we collected a high‐SNR reference data at b = 6000 ${\text{s/mm}}^{2}$ using a 2D single‐shot axial acquisition at 1 mm isotropic resolution. This reference data was generated using 44 averages and the same 22 gradient directions as the 3D acquisition (scan time was 33 min for a coverage of 20 mm). The comparison given in Figure S10 confirms that the diffusion contrast generated in the 3D‐qModeL reconstruction reflects the true diffusion contrast as measured using a 2D acquisition employing traditional reconstruction.

An Additional set of experiments was also performed to evaluate the level of spatial blurring present in the 3D‐qModeL reconstructed maps. For this purpose, high‐SNR reference data at 1 mm isotropic resolution at b1000 was collected using 2D axial acquisition with 22 averages and 22 directions, with matched FOV and slice positioning as the 3D data (scan time was 17 min for a coverage of 20 mm). A 1.5 mm isotropic resolution data set was also collected from the same session. The FA maps generated from the above datasets (after denoising with MPPCA) are provided in Supporting Information Figure S11. The comparison serves to establish the additional spatial details present in an isotropic 1 mm data vs. 1.5 mm data. Next, we performed a second scan where 3D data was also collected along with the 2D 1 and 1.5 mm data. We show the comparison of the FA maps from all three cases in Supporting Information Figure S12. (The protocol details for the above experiments are also added in the Supporting Information Table S3). We note that 3D‐qModeL reconstruction indeed preserves the high‐resolution features that are seen in the 2D high‐SNR reference data, despite being prone to spatial blurring from motion (and a lower SNR compared to the 2D data). However, we also note that a major limitation of the current qModeL formulation is that it is highly susceptible to spatial blurring due to motion between the scans. This source of spatial blurring is due to the absence of a motion‐compensation step within the reconstruction process, the incorporation of which can further improve the spatial fidelity of the qModeL reconstructed images.

## 4 DISCUSSION

Our main goal in this work is to demonstrate the utility of the accelerated 3D‐msDWI to support advanced microstructure modeling in practical in‐vivo applications. In the past, 3D‐msDWI studies requiring extended k‐q space coverage have been primarily limited to preclinical applications.^37^ The high VAT inherent to traditional fully‐sampled 3D‐msDWI precluded its application for in‐vivo studies. However, a reduced‐VAT implementation of 3D‐msDWI can trade the long scan time to cover more q‐space, which makes it a viable candidate for advanced in‐vivo human studies. In this work, we demonstrate that the high SNR‐efficiency of 3D‐msDWI can enable unique applications such as high b‐value high‐resolution studies, a scenario where the low SNR‐efficiency of 2D methods requires sacrificing either the resolution or coverage. Specifically, we demonstrated the feasibility of deriving robust parameter fits of the 3‐compartment model at 1 mm isotropic resolution from a 13‐min scan. We tested the practical feasibility of the highly accelerated experiment by performing the study on older and younger populations.

The above‐demonstrated feasibility of 3D‐msDWI for extended k‐q sampling relies on an under‐sampled acquisition‐reconstruction framework. High redundancy in the 6D k‐q space allows massive under‐sampling, and 3D acquisitions enable under‐sampling along both phase‐encoding directions. Using a 2D ky‐kz CAIPI pattern with randomized k‐q under‐sampling, we achieved a 10‐fold acceleration to enable the study. The sparsely sampled joint k‐q data were reconstructed using the 3D‐qModeL reconstruction that relies on a DL‐prior to recover the missing k‐q samples. Here, the accuracy of the trained DL‐prior is essential to ensure reliable signal reconstruction and accurate parametric maps. Because the b6000 shell has a low signal amplitude, special care is required during training to recover the signal from this shell during the joint recovery. Relying on a simple MSE loss causes the training to favor high‐SNR (low‐b) data and neglect low‐SNR (high‐b) data, compromising reconstruction quality, especially of intra‐axonal parameters, which are sensitive to high‐b signals. To address this, we re‐weighted the loss function to emphasize high‐b shell data. A careful analysis of the intra‐axonal parameters in Figures 5, 6, 7, 8, along with the DTI and DKI parameters, confirms that the quality of the parameter maps from the 3D‐qModeL reconstruction matches that of the 2D methods. The intra‐axonal parameter fits obtained from the ablated qModeL reconstruction, along with those of a non‐weighted MSE loss, are provided in Supporting Information (Figures S13–S14), which demonstrates the crucial role of the learned prior in the resulting parameter fit validity.

We employed two separate acquisitions to validate the parameter maps obtained from the accelerated 3D‐msDWI method with the 3D‐qModeL reconstruction. In the first case, 2D‐SDE datasets with matched q‐space coverage were utilized. In the second case, 2D‐MDE datasets were utilized with more extensive q‐space coverage and a richer encoding of the diffusion process. Both datasets were matched for scan time and coverage. As a result, the 2D‐SDE data allows multiple imaging averages (NEX = 3) and a shorter TE, while the 2D‐MDE data samples a richer encoding space with more directional averages, albeit with a longer TE. Some disparity is noticeable in the parameter maps generated using the SDE and MDE data, and mainly arises from two reasons: (i) the MDE and SDE encoding provides different information about the microstructure (accounted using the parameter β in the kernel) and (ii) the microstructure parameter estimation depends on the SNR. In the absence of a ground truth, it is not clear if MDE provides more accurate results compared to SDE or vice‐versa, although literature supports that non‐SDE data improves the accuracy of certain microstructural maps using simulations.^19^ While validation of the various encoding methods is beyond the scope of this work, our analysis makes clear that in the WM‐ROIs studied, the parameters generated by the 3D‐msDWI framework are within the expected range predicted by the 2D‐SDE and the 2D‐MDE. Additionally, our measured $D_{a}$ values from the 2D and 3D acquisitions range 2.1–2.3 and 2–2.5 $\mu m^{2}/ms$, respectively, and agree with previously reported values in human WM (2–2.4 $\mu m^{2}/ms$).^6^, ^38^ Given that SNR plays a crucial role in the parameter accuracy,^19^, ^39^ any benefits from improved SNR of the 3D‐SDE and 2D‐SDE on HPG systems may need to be studied further in future studies.

Table S3 in the supporting information lists the acquisition details for generating a 2D‐based high‐SNR reference data at 1mm for b‐values of 1000 and 6000 s/mm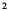. From the table, it is evident that it will take 51/99 min of scan time to acquire a high‐SNR reference data with 3 shells and 66 directions using the 2D methods at 1 mm resolution, even for a limited coverage of 20 mm. Due to the long scan time involved, we have refrained from using such reference data in the current study. Nevertheless, through several other experiments, including the subject‐level and cross‐subject ROI‐based analysis, we have provided sufficient evidence to confirm that the parameter estimates from the 3D‐qModeL reconstruction are in the expected range noted in healthy subjects and are in agreement with the values reported in the literature. Furthermore, simulation‐based signal reconstructions using independently estimated model parameters are also noted to follow the qModeL reconstruction closely (see Supporting Information, Figure S9), providing additional validation.

We note that inadequate fat suppression has resulted in spurious signals in the current implementation of our 3D pulse sequence, which has contributed to errors in the parameter estimation of the 3D data. Future works will explore improved fat suppression methods to minimize this artifact.

## 5 CONCLUSIONS

Our study demonstrates the extended capabilities of 3D‐msDWI for high b‐value studies and its feasibility for in‐vivo applications at high resolutions. We demonstrate that the SNR‐efficiency of the 3D‐msDWI can be harvested to support advanced microstructure studies that require extensive k‐q coverage.

## Supporting information




Data S1: Supporting Information
**Figure S1:** Phase compensation example from subject 2. To evaluate the efficacy of the phase‐compensated reconstruction, the corresponding 2D‐SDE images and their denoised versions are provided. Although the distortions in the 2D SDE denoised images are not perfectly matched, they still serve to confirm that phase compensation in the 3D‐msDWI method is adequate. Note that the displayed FOV of the 3D and the 2D datasets are different due to 3D being an axial scan and 2D being a sagittal scan.
**Figure S2:** The 3D‐qModeL joint reconstruction (top) followed by the slab combination to generate whole‐brain volumes (bottom), is shown from two subjects.
**Figure S3:** FA, MD, and MK maps computed for the various acquisitions from a second subject.
**Figure S4:** Three‐compartment model parameter maps shown from subject #2.
**Figure S5:** Plots of the distribution of $D_{a}$ values in the white matter ROIs from all 9 subjects. Here, blue, red, and yellow colors represent 2D‐SDE, 2D‐MDE, and 3D‐SDE, respectively.
**Figure S6:** Plots of the distribution of $f_{a}$ values in the white matter ROIs from all 9 subjects. Here, blue, red, and yellow colors represent 2D‐SDE, 2D‐MDE, and 3D‐SDE, respectively.
**Figure S7:** The 2D‐SENSE, the denosing of the 2D‐SENSE using the DAE, and the qModeL reconstruction shown from a slice with the same diffusion gradient applied at b‐values of 1000, 2000, and 6000 s/${\text{mm}}^{2}$, all shown in their native image colorscale.
**Figure S8:** A zoomed region from the noisy 2D‐SENSE reconstruction is shown (b = 6000 s/${\text{mm}}^{2}$). The red and orange arrows are placed where the voxel exhibits a perceived high and low SNR, respectively. The voxel plots from the two arrows are shown on the left side. The red plot shows the noisy 2D‐SENSE signal, with the red dotted vertical line indicating the diffusion direction shown in the 2D‐SENSE and the qModeL images. With the red voxel being the reference, the voxel signal plot indicates that the 2D‐SENSE signal is noisy at the orange voxel, which is denoised to the expected level by the DAE‐denoised and the qModeL reconstruction, as indicated by the green and the blue plots, respectively.
**Figure S9:** The signal simulated at the orange voxel location based on the estimated model parameters agrees with the denoised and the qModeL reconstructed signals. Simulations were performed using parameters obtained from the MDE data (left) of the same subject and 3D SDE data (right).
**Figure S10:** 5 DWIs from the b6000 shell of qModeL reconstruction from a given subject are shown on the top in the native color bar. A separate scan was performed on the same subject at 1 mm isotropic resolution with a b‐value of 6000 s/${\text{mm}}^{2}$, using 2D acquisition with matched diffusion directions (shown in the middle row), to confirm that the qModeL diffusion contrast matches the diffusion contrast of the high‐SNR reference data. The imaging parameters of the reference data are given in Table S3. The simulated diffusion contrast corresponding to a b‐value of 6000 s/${\text{mm}}^{2}$ for the 5 directions is also shown in the bottom row.
**Figure S11:** New 2D data at b1000 collected with 22 averages at 1 mm isotropic resolution (left) shows more anatomical details in their corresponding FA maps compared to the FA map of the 1.5 mm data (same subject and same session). The main differences are in the fine structures adjacent to the major WM bundles, as indicated by the arrow.
**Figure S12:** The FA maps from the second set of b1000 data collected. In this scan session, 3D data were also collected at 1 mm isotropic resolution and reconstructed using 3D‐qModeL (FA maps shown on the left), along with 2D data at 1.5 mm isotropic resolution (FA maps shown in the middle), and the 2D data at 1 mm isotropic resolution (FA maps shown on the right). The 1.5 and 1 mm 2D were acquired using 1 and 22 averages, respectively, and were denoised using MPPCA. Note that the accelerated 3D‐msDWI only involves 6 TRs and hence should theoretically have a lower SNR than the 2D data with 22 averages. More details about the imaging parameters are given in Table S3.
**Figure S13:** The fitted parameters of the intra‐axonal compartment from the ablated qModeL reconstruction with no q‐space manifold prior (shown from the same slice as in Figure 6).
**Figure S14:** The intra‐axonal diffusivity estimates from a weighted MSE loss (left) and a non‐weighted MSE loss (right).
**Table S1:** Imaging Parameters of the 3D‐SDE, 2D‐SDE and 2D‐MDE datasets.
**Table S2:** DAE MSE losses for the different weights studied.
**Table S3:** Details of the additional experiments performed.
