# Supplementary material for: Advanced microstructure imaging at high b‐values and high resolution combining ultra‐high performance gradient diffusion imaging and model‐based deep learning demonstrated using 3D multi‐slab acquisition
Source: Magn Reson Med. 2025 Aug 24;95(1):299–313. doi: 10.1002/mrm.70046 (PMC12620157; doi:10.1002/mrm.70046)
Supplement: Supplementary file 1 — Data S1: Supporting Information Figure S1: Phase compensation example from subject 2. To evaluate the efficacy of the phase‐compensated reconstruction, the corresponding 2D‐SDE images and their denoised versions are provided. Although the distortions in the 2D SDE denoised images are not perfectly matched, they still serve to confirm that phase compensation in the 3D‐msDWI method is adequate. Note that the displayed FOV of the 3D and the 2D datasets are different due to 3D being an axial scan and 2D being a sagittal scan. Figure S2: The 3D‐qModeL joint reconstruction (top) followed by the slab combination to generate whole‐brain volumes (bottom), is shown from two subjects. Figure S3: FA, MD, and MK maps computed for the various acquisitions from a second subject. Figure S4: Three‐compartment model parameter maps shown from subject #2. Figure S5: Plots of the distribution of Da values in the white matter ROIs from all 9 subjects. Here, blue, red, and yellow colors represent 2D‐SDE, 2D‐MDE, and 3D‐SDE, respectively. Figure S6: Plots of the distribution of fa values in the white matter ROIs from all 9 subjects. Here, blue, red, and yellow colors represent 2D‐SDE, 2D‐MDE, and 3D‐SDE, respectively. Figure S7: The 2D‐SENSE, the denosing of the 2D‐SENSE using the DAE, and the qModeL reconstruction shown from a slice with the same diffusion gradient applied at b‐values of 1000, 2000, and 6000 s/mm2, all shown in their native image colorscale. Figure S8: A zoomed region from the noisy 2D‐SENSE reconstruction is shown (b = 6000 s/mm2). The red and orange arrows are placed where the voxel exhibits a perceived high and low SNR, respectively. The voxel plots from the two arrows are shown on the left side. The red plot shows the noisy 2D‐SENSE signal, with the red dotted vertical line indicating the diffusion direction shown in the 2D‐SENSE and the qModeL images. With the red voxel being the reference, the voxel signal plot indicates that the 2D‐SENSE signal is noisy at the oran [file MRM-95-299-s001.pdf]

Supporting Information

A. 3-compartment model details

The 3-compartment model given in Eq 1 models a mesoscopic WM voxel to consist of an intra-axonal compartment, an extra-axonal compartment and a cerebrospinal fluid (CSF) compartment. The predicted diffusion signal for an applied gradient direction and b-value, $S(\mathbf{g}, b)$, is given by the spherical convolution of the fiber orientation distribution, $\mathcal{P}(\mathbf{n})$, with the microstructure kernel $K(b, \mathbf{g} \cdot \mathbf{n})$ as follows :(1, 2)

$$S(b, \mathbf{g}) = S_0 \int_{\mathbf{n}} d\mathbf{n}\mathcal{P}(\mathbf{n}) \circledast \mathbf{K}(\mathbf{b}, \mathbf{g} \cdot \mathbf{n}) \quad \text{where} \qquad [1]$$

$$K(b, \mathbf{g} \cdot \mathbf{n}) = \underbrace{f_{\mathrm{a}} e^{-bD_a \mathbf{g} \cdot \mathbf{n}}}_{\text{intra-axonal}} + \underbrace{f_{\mathrm{e}}\ e^{-bD_e^{\perp} - b(D_e^{\|} - D_e^{\perp}) \mathbf{g} \cdot \mathbf{n}}}_{\text{extra-axonal}} + \underbrace{f_{\mathrm{w}} e^{-bD_{\mathrm{w}}}}_{\text{CSF}}$$

where $\mathbf{n}$ denotes the fiber direction and S_0 is the non-diffusion-weighted reference signal. The parameters of the model include the intra-axonal volume fraction, f_a and diffusivity D_{a}; the extra-axonal volume fraction f_{e} and its parallel and perpendicular diffusivities, $D_e^{\|}$ and $D_e^{\perp}$, and the parameters of the CSF compartment: f_{w} and D_{w}. Except for D_{w} which is fixed at $3\times\ 10^{-3}$ mm^2/sec, the other kernel parameters and $\mathcal{P}$, are estimated from the measured multi-shell data under the constraint that $f_a + f_e + f_w = 1$. Solving for these parameters using non-linear least squares (NNLS) can result in degenerate solutions leading to wrong parameter estimates at the clinical MRI SNR regime. Recently, supervised machine learning-based approaches have been used to solve these parameters to avoid degenerate solutions (3) and generate more accurate estimates than those obtained using the NNLS method (4–6).

Table S1: Imaging Parameters of the 3D-SDE, 2D-SDE and 2D-MDE datasets

Dataset :	3D-SDE
Slab FOV	20mm
FOV_{xy}(Axial)	216mm x 216mm
N_{k_z}	20
Matrix size (3D)	216 x 216 x 20
Spatial Resolution (mm)	1 x 1 x 1
# slabs	8
Axial coverage	112 mm
# k_y shots	3
Partial Fourier ky	0.7
TE1, TE2	52 ms, 116ms
TR	2 sec

Dataset:	2D-SDE	2D-MDE
# Slices	100	100
FOV (Sagittal)	216mm x 216mm	216mm x 216mm
Slice Thickness	1.5 mm	1.5 mm
Matrix size (2D)	144 x 144	144 x 144
Spatial Resolution (mm)	1.5 x 1.5 x 1.5	1.5 x 1.5 x 1.5
# k_y shots	1	1
Partial Fourier ky	0.7	0.7
TE	49.7 ms	70.6 ms
TR	3.3 sec	5.8 sec
Multi-band Factor	3	3
Averages	3	1

Table S2: DAE MSE losses for the different weights studied

Total MSE loss= $\text{loss}^{shell1} \times \text{weight}_1 + \text{loss}^{shell2} \times \text{weight}_2 + \text{loss}^{shell6} \times \text{weight}_6$

Total MSE Loss	loss^{shell1}, weight_1	loss^{shell2}, weight_2	loss^{shell6}, weight_6
0.00177	0.000491, 1.0	0.000473, 1.0	0.000806, 1.0
0.002055	0.000475, 1.0	0.000420, 1.0	0.000772, 1.5
0.002777	0.000683, 1.0	0.000533, 1.0	0.000780, 2.0

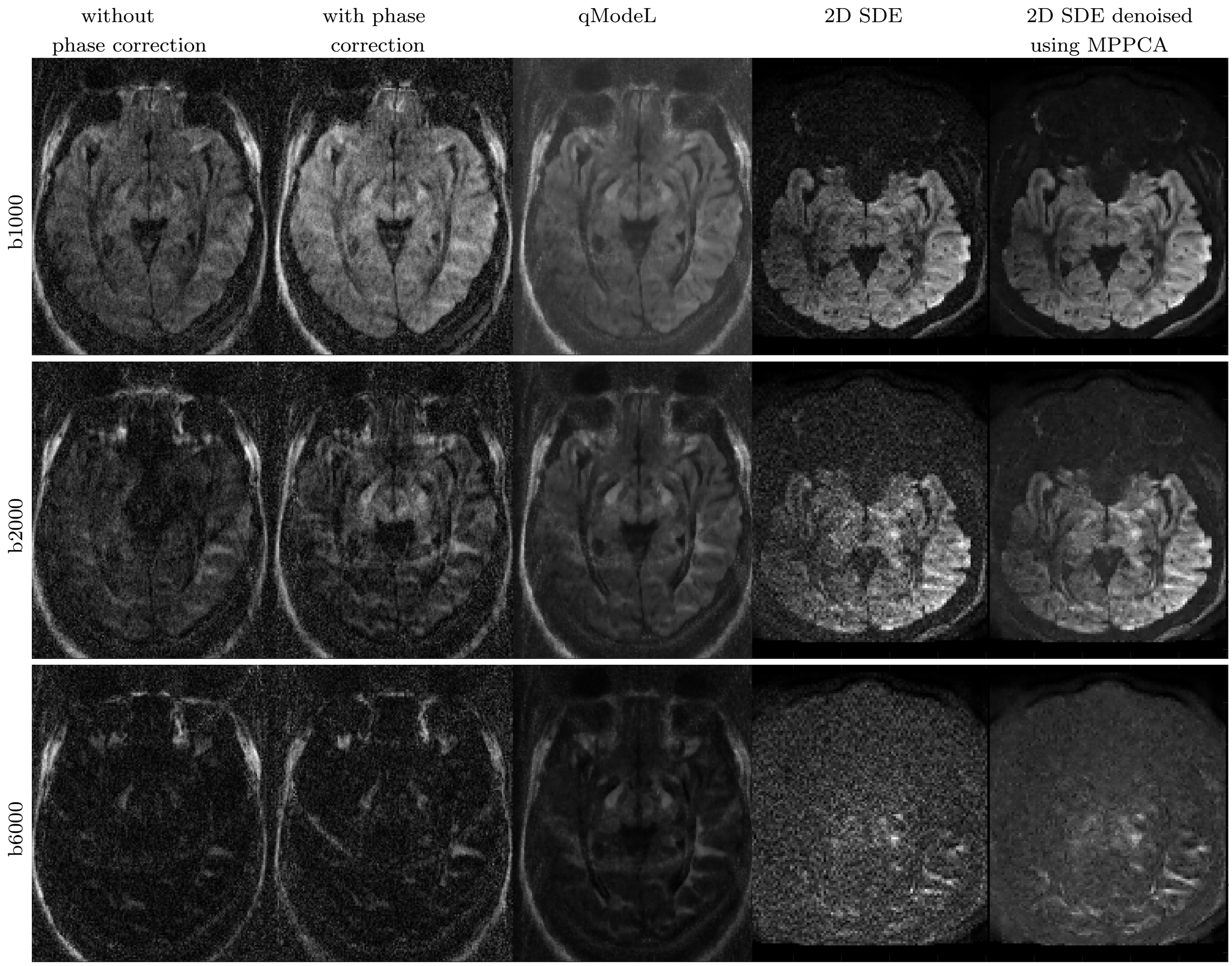

Figure S1: Phase compensation example from subject 2. To evaluate the efficacy of the phase compensated reconstruction, the corresponding 2D-SDE images and their denoised versions are provided. Although the distortions in the 2D SDE denoised images are not perfectly matched they still serve to confirm that phase compensation in the 3D-msDWI method is adequate. Note that the displayed FOV of the 3D and the 2D datasets are different due to 3D being an axial scan and 2D being a sagittal scan.

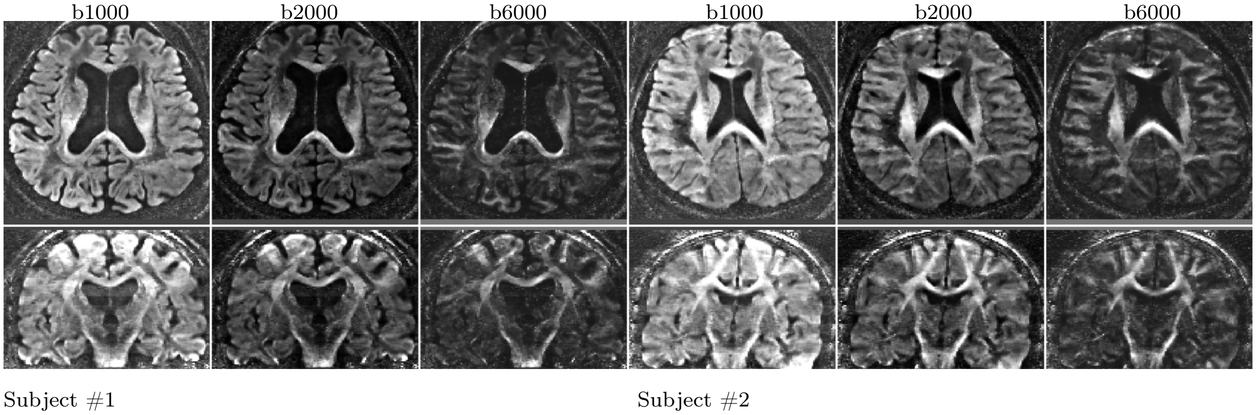

Figure S2: The 3D-qModeL joint reconstruction (top) followed by the slab combination to generate whole-brain volumes (bottom), are shown from two subjects.

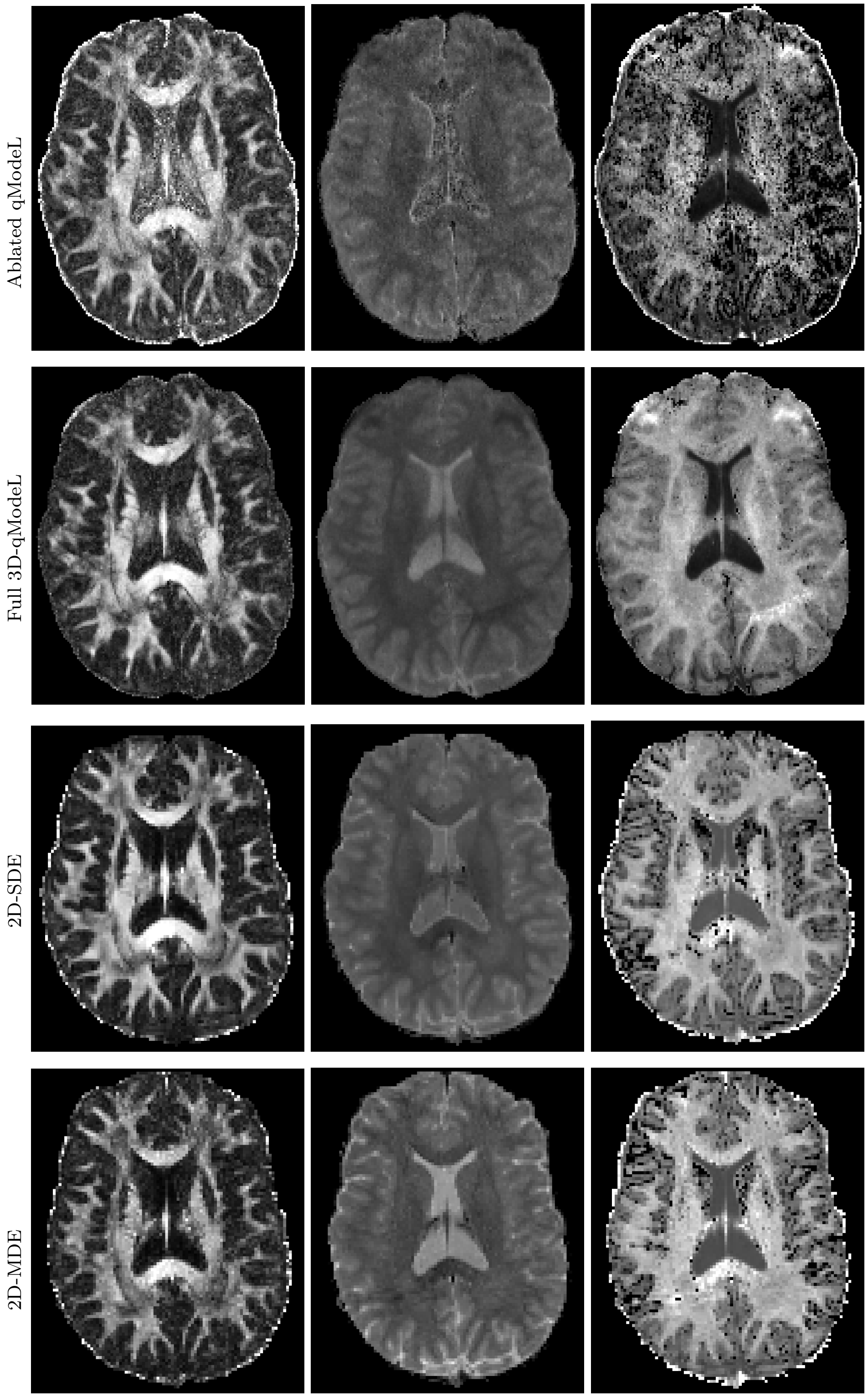

Figure S3: FA, MD and MK maps computed for the various acquisitions from a second subject.

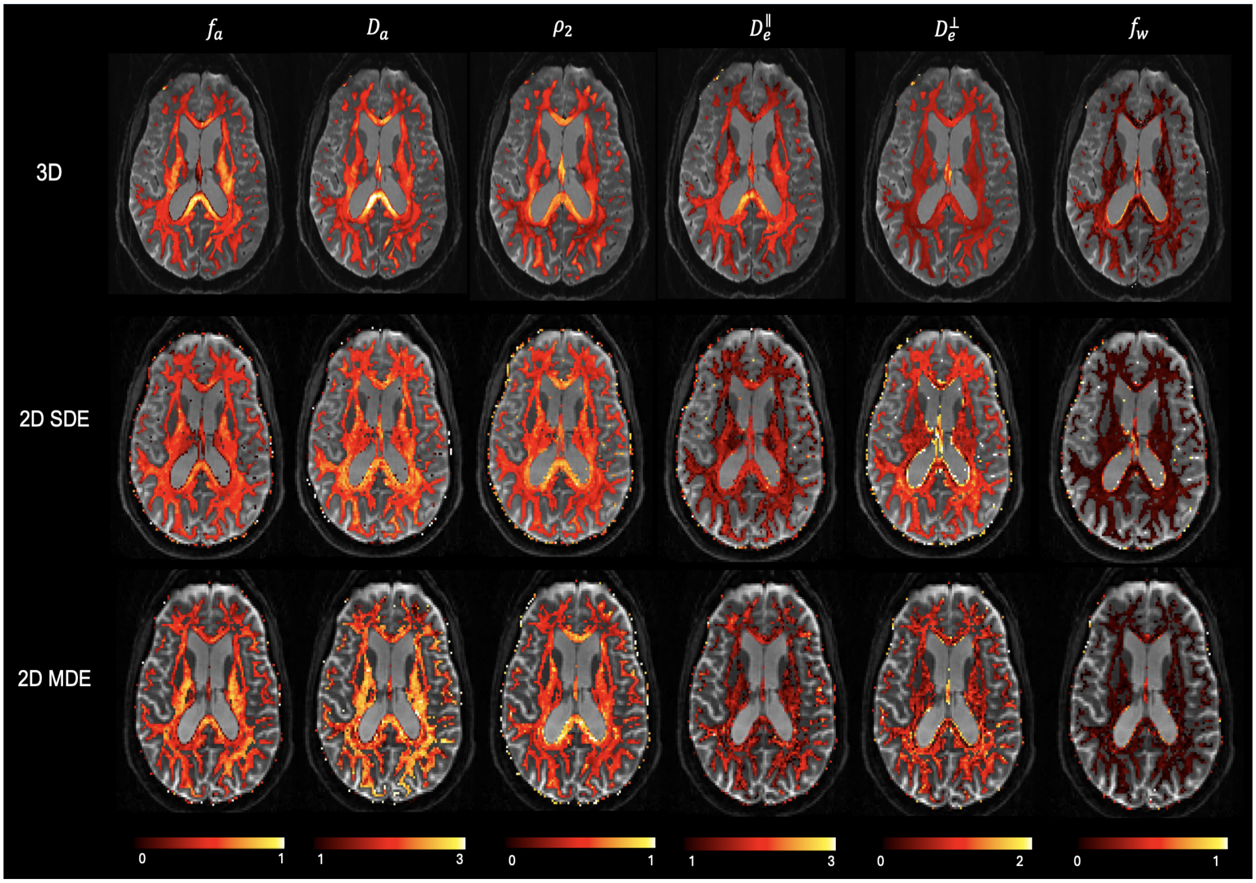

Figure S4: Three-compartment model parameter maps shown from subject #2.

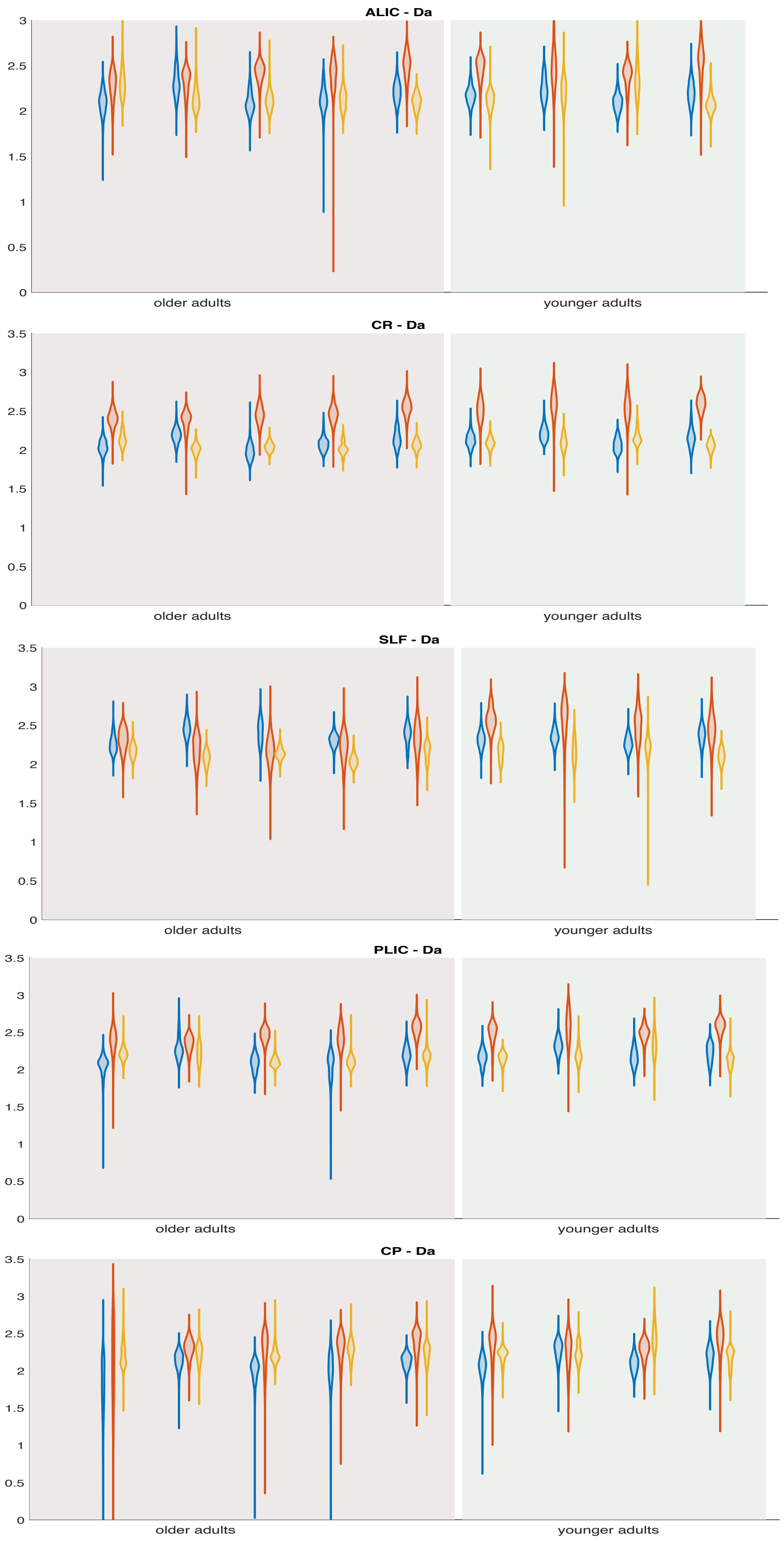

Figure S5: Plots of the distribution of D_a values in the white matter ROIs from all 9 subjects. Here blue, red and yellow colors represent 2D-SDE, 2D-MDE and 3D-SDE respectively.

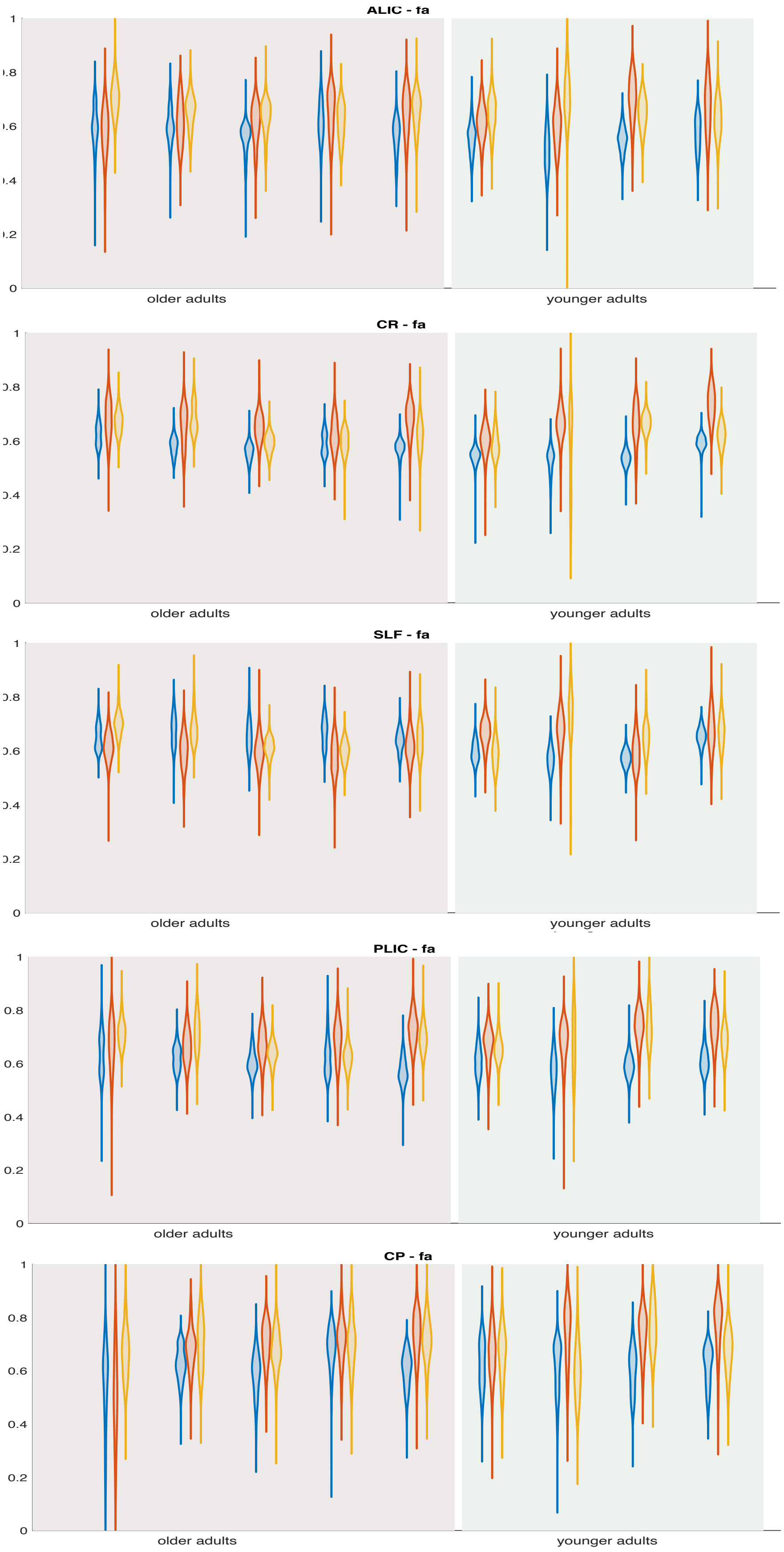

Figure S6: Plots of the distribution of f_a values in the white matter ROIs from all 9 subjects. Here blue, red and yellow colors represent 2D-SDE, 2D-MDE and 3D-SDE respectively.

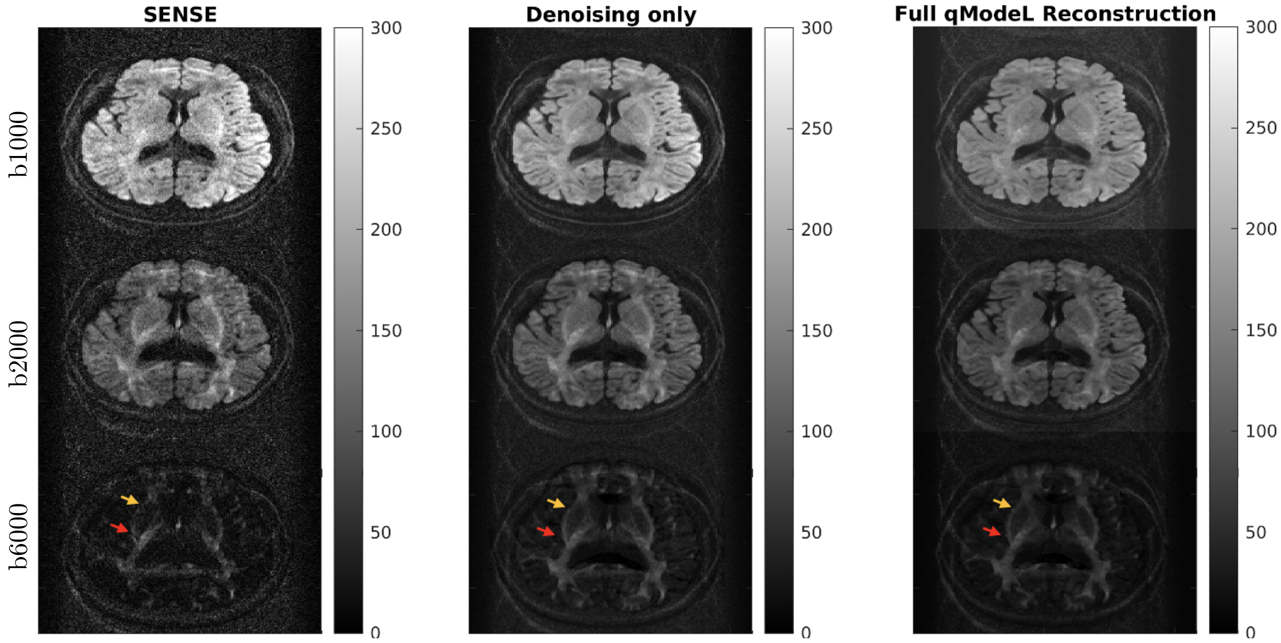

Figure S7: The 2D-SENSE, the denosing of the 2D-SENSE using the DAE, and the qModeL reconstruction shown from a slice with the same diffusion gradient applied at b-values of 1000, 2000 and 6000s/mm^2, all shown in their native image colorscale.

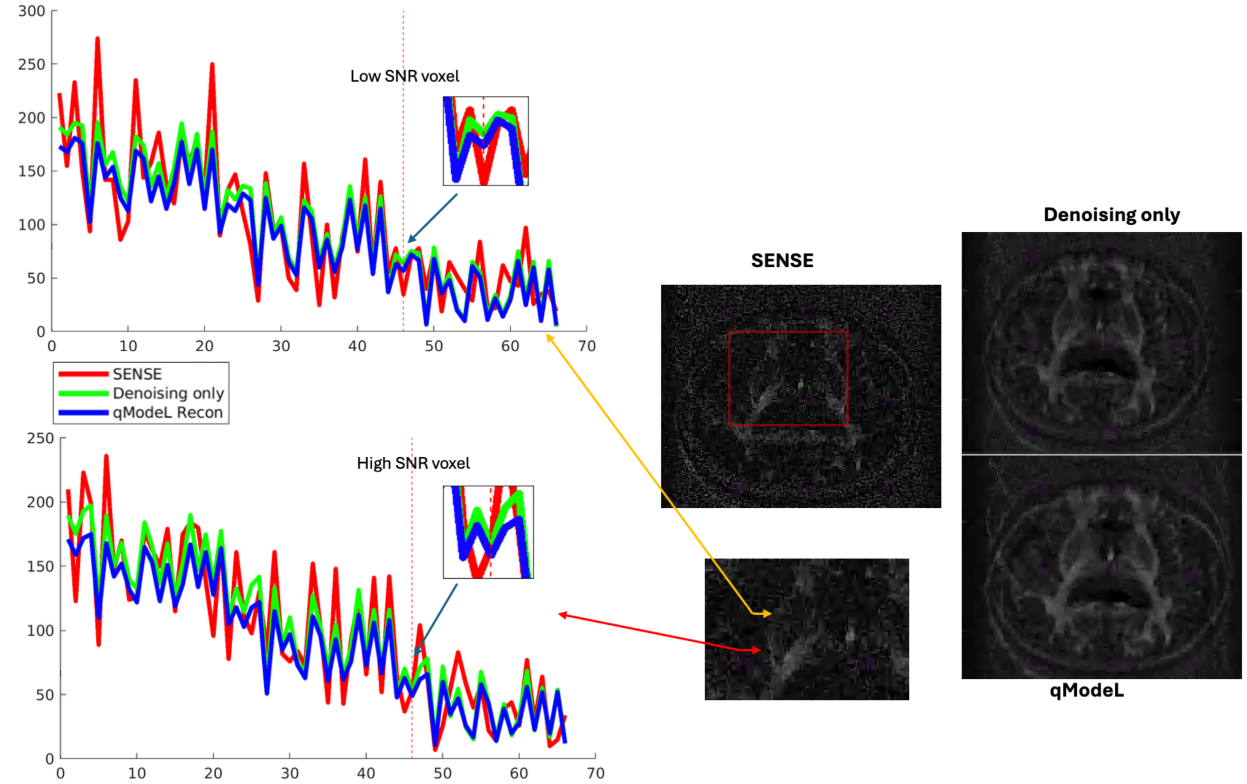

Figure S8: A zoomed region from the noisy 2D-SENSE reconstruction is shown (b=6000s/mm^2). The red and orange arrows are placed where the voxel exhibit a perceived high and low SNR respectively. The voxel plots from the two arrows are shown on the left side. The red plot shows the noisy 2D-SENSE signal, with the red dotted vertical line indicating the diffusion direction shown in the 2D-SENSE and the qModeL images. With the red voxel being the reference, the voxel signal plot indicates that the 2D-SENSE signal is noisy at the orange voxel, which is denoised to the expected level by the DAE-denoised and the qModeL reconstruction, as indicated by the green and the blue plots respectively.

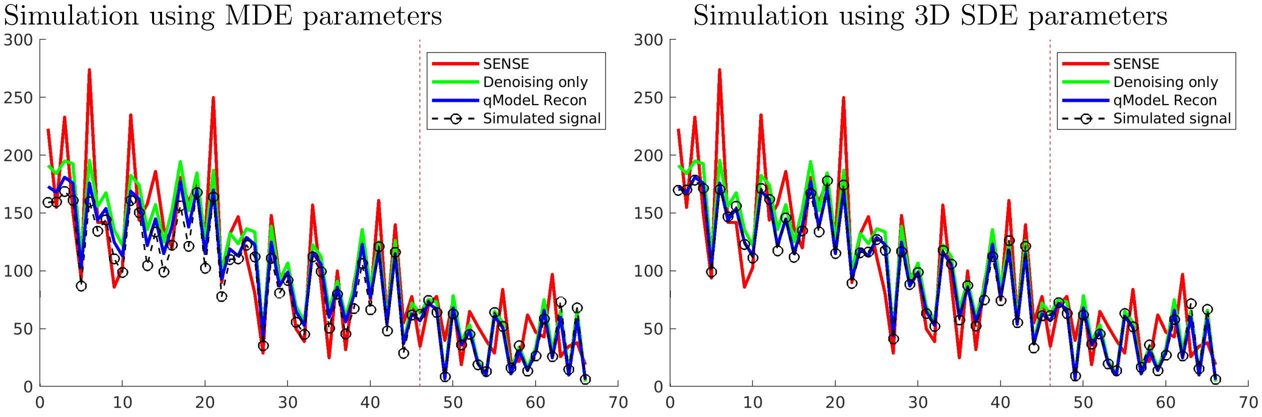

Figure S9: The signal simulated at the orange voxel location based on the estimated model parameters agrees with the denoised and the qModeL reconstructed signals. Simulations were performed using parameters obtained from the MDE data (left) of the same subject and 3D SDE data (right)

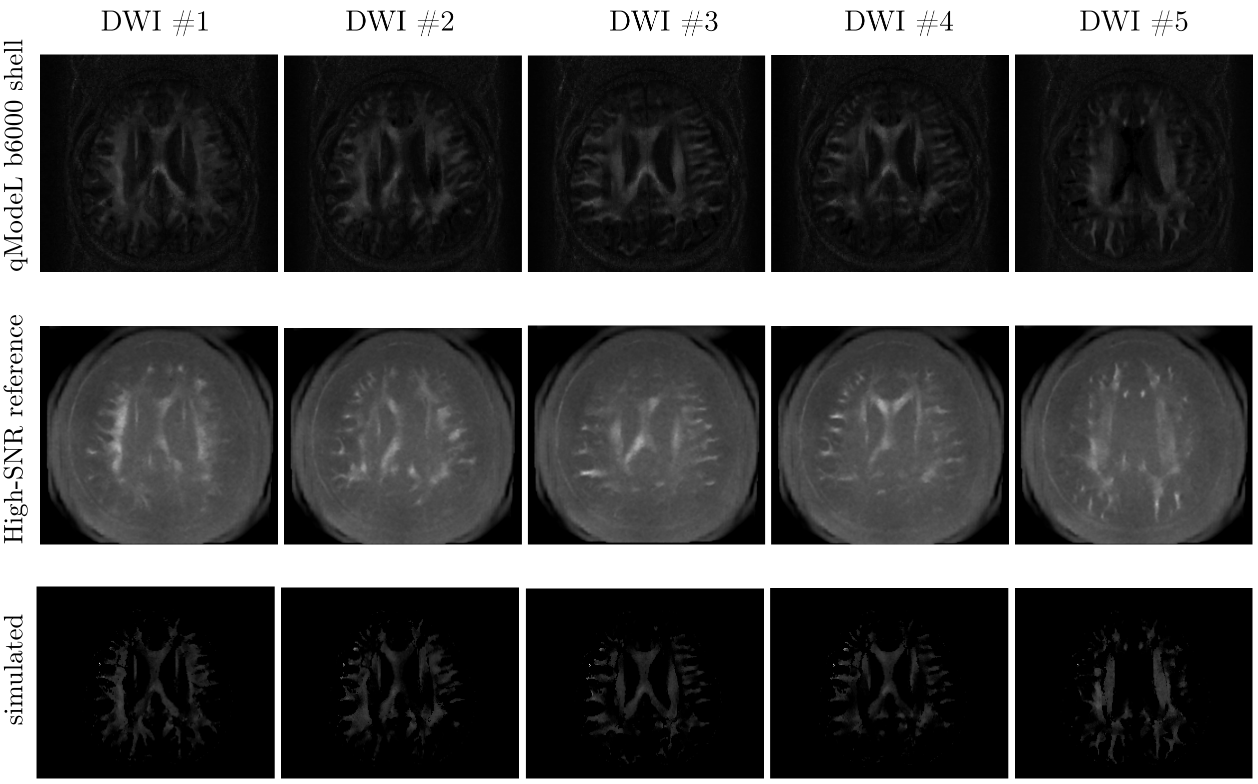

Figure S10: 5 DWIs from the b6000 shell of qModeL reconstruction from a given subject is shown on the top in the native color bar. A separate scan was performed on the same subject at 1mm isotropic resolution for b-value of $6000s/mm^2$ using 2D acquisition with matched diffusion directions (shown at the middle row) to confirm that the qModeL diffusion contrast matches with the diffusion contrast of the high-SNR reference data. The imaging parameters of the reference data are given in Table S3. The simulated diffusion contrast corresponding to b-value of $6000s/mm^2$ for the 5 directions is also shown in the bottom row.

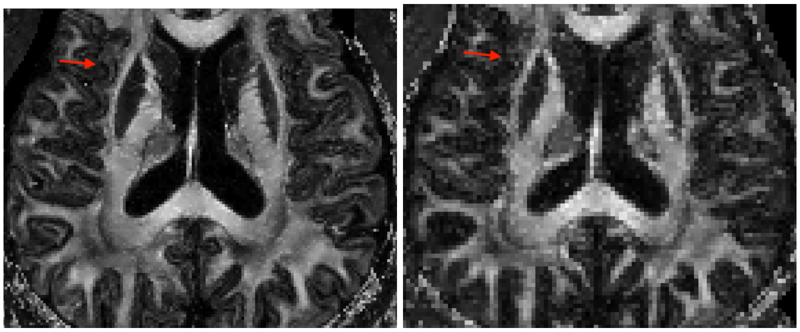

Figure S11: New 2D data at b1000 collected with 22 averages at 1mm isotropic resolution (left) shows more anatomical details in their corresponding FA maps compared to the FA map of the 1.5mm data (same subject and same session). The main differences are in the fine structures adjacent to the major WM bundles as indicated by the arrow.

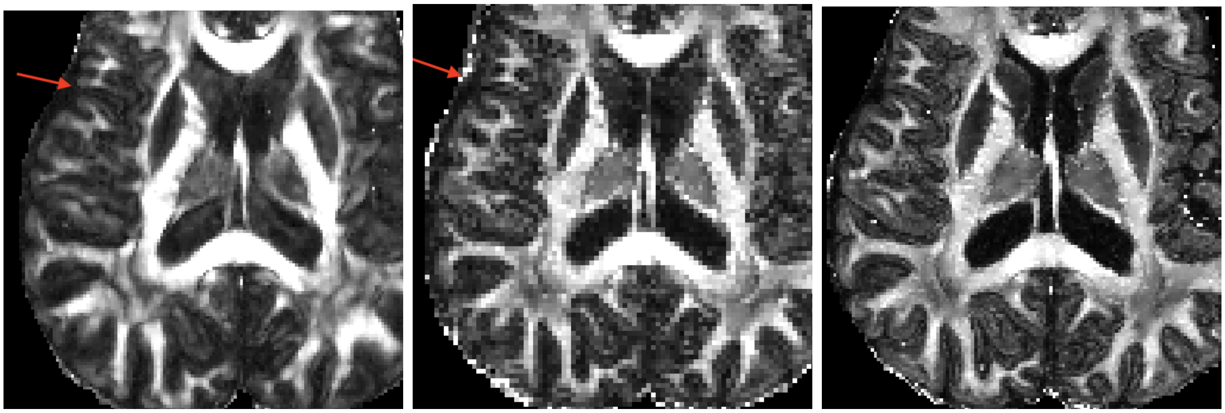

Figure S12: The FA maps from the second set of b1000 data collected. In this scan session, 3D data was also collected at 1mm isotropic resolution and reconstructed using 3D-qModeL (FA maps shown on left), along with 2D data at 1.5mm isotropic resolution (FA maps shown in the middle), and the 2D data at 1mm isotropic resolution (FA maps shown on the right). The 1.5mm and 1mm 2D were acquired using 1 and 22 averages respectively and were denoised using MPPCA. Note that the accelerated 3D-msDWI only involves 6 TRs and hence should theoretically have a lower SNR than the 2D data with 22 averages. More details about the imaging parameters are given in Table S3.

Table S3: Details of the additional experiments performed.

Dataset	Imaging Resolution	TR per direction	Directions	Averages/ # kz encodes	Spatial coverage	scan-time
2D b6000	1mm	2 sec	22	44	20 mm	33 minutes
2D b1000	1mm	2 sec	22	22	20 mm	17 minutes
3D-msDWI (b1000, 2000, 6000)	1mm	2 sec	66	6	112mm	13.5 minutes

f_a fit from ablated qModeL case D_a fit from the ablated qModeL case

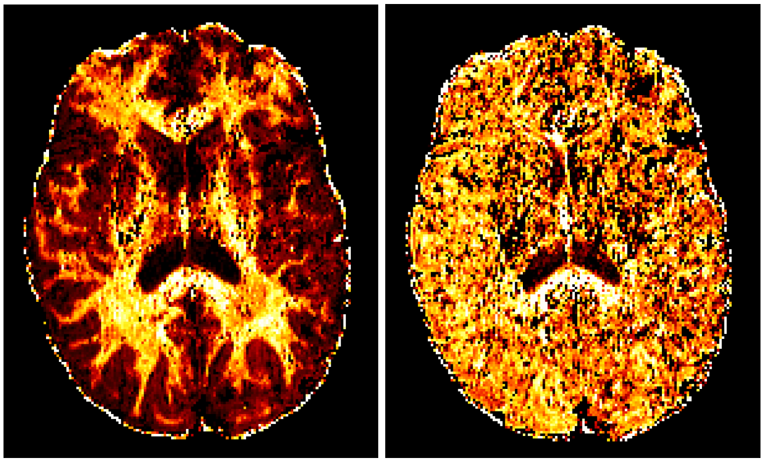

Figure S13: The fitted parameters of the intra-axonal compartment from the ablated qModeL reconstruction with no q-space manifold prior (shown from the same slice as in figure 6).

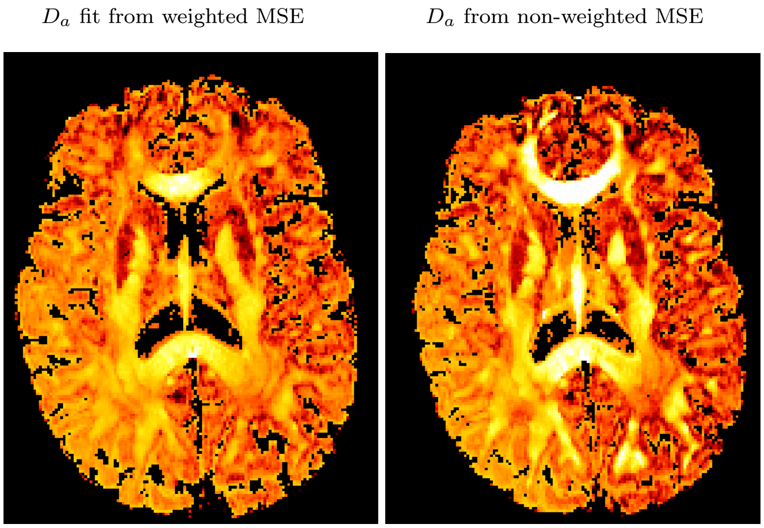

Figure S14: The intra-axonal diffusivity estimates from a weighted MSE loss (left) and a non-weighted MSE loss (right).

References

1 Novikov DS, Jespersen SN, Kiselev VG, and Fieremans E. Quantifying Brain Microstructure with Diffusion MRI: Theory and Parameter Estimation. *arxiv.org*, 2016.

2 Novikov DS, Veraart J, Jelescu IO, and Fieremans E. Rotationally-Invariant Mapping of Scalar and Orientational Metrics of Neuronal Microstructure with Diffusion MRI. *NeuroImage*, 2018.

3 Jelescu IO, Veraart J, Fieremans E, and Novikov DS. Degeneracy in Model Parameter Estimation for Multi-Compartmental Diffusion in Neuronal Tissue. *NMR in Biomedicine*, 2016.

4 Reisert M, Kellner E, Dhital B, Hennig J, and Kiselev VG. Disentangling Micro from Mesostructure by Diffusion MRI: A Bayesian Approach. *NeuroImage*, 147:964–975, 2017.

5 Coelho S, Baete SH, Lemberskiy G, Ades-Aron B, Barrol G, Veraart J, Novikov DS, and Fieremans E. Reproducibility of the Standard Model of Diffusion in White Matter on Clinical MRI Systems. *NeuroImage*, 257, 2022.

6 Coelho S, Fieremans E, and Novikov D. How do we know we measure tissue parameters, not the prior? *ISMRM*, 0397, 2022.
